# Association between trauma and socioeconomic deprivation: a registry-based, Scotland-wide retrospective cohort study of 9,238 patients

**DOI:** 10.1186/s13049-016-0275-7

**Published:** 2016-07-07

**Authors:** Alasdair R. Corfield, Danny F. MacKay, Jill P. Pell

**Affiliations:** Emergency Department, Royal Alexandra Hospital, Paisley, PA2 9PN UK; Institute of Health and Wellbeing, University of Glasgow, Glasgow, G12 8RZ UK

**Keywords:** Trauma, Deprivation, Inequalities, Registry

## Abstract

**Background:**

Trauma remains a leading cause of morbidity and mortality in the UK and throughout the world. Socioeconomic deprivation has been linked with many types of ill-health and previous studies have shown an association with injury in other parts of the world. The aim of this study was to investigate the association between socioeconomic deprivation and trauma incidence and case-fatality in Scotland.

**Methods:**

The study included nine thousand two hundred and thirty eight patients attending Emergency Departments following trauma across Scotland in 2011-12. A retrospective cohort study was conducted using secondary data extracted from the national trauma registry. Postcode of residence was used to generate deciles using the Scottish Index of Multiple Deprivation. The incidence rate ratio (IRR) was calculated to allow comparison of incidence of trauma across SIMD deciles. For mortality, observed: expected ratios were obtained using observed mortality in the cohort and expected deaths using probability of survival based on Trauma and Injury Severity Score (TRISS) method.

**Results:**

Compared with the most deprived decile, the least deprived had an incidence rate ratio (IRR) for all trauma of 0.43 (95 % CI 0.32–0.58, *p* < 0.001). The association was stronger for penetrating trauma (IRR 0.07, 95 % CI .01–0.56, *p* = 0.011). There was a significant interaction between age, gender and SIMD. For case fatality, multivariate logistic regression showed that, severity of trauma (ISS > 15) OR 18.11 (95 % CI 13.91 to 23.58) and type of injury (Penetrating versus blunt injury) OR 2.07 (95 % CI 1.15 to 3.72) remain as independent predictors of case fatality in this dataset.

**Discussion:**

Our data shows a higher incidence of trauma amongst a socioeconomically deprived population, in keeping with other areas of the world. In our dataset, outcome, as measured by in-hospital mortality, does not appear to be associated with socioeconomic deprivation.

**Conclusion:**

In Scotland, populations living in socioeconomically deprived areas have a higher incidence of trauma, especially penetrating trauma, requiring hospital attendance. Case fatality is associated with more severe trauma and penetrating trauma, but not socioeconomic deprivation.

**Electronic supplementary material:**

The online version of this article (doi:10.1186/s13049-016-0275-7) contains supplementary material, which is available to authorized users.

## Background

Injuries due to trauma remain a major cause of morbidity and mortality in the UK and throughout the world [1]. It is the leading cause of death in the 15–44 year age-group worldwide, [2] and a major cause of disability and loss of earnings [3]. Because the incidence of trauma is particularly high in the younger population, a mean of 36 life years are lost for each death due to trauma [4]. The health impact of trauma and injuries has been recognised by UK Government health policy over the past 30 years [5, 6]. As well as the considerable health impact, trauma and injuries have a considerable economic impact. Direct health costs in the UK are estimated at £400 million per annum, and the total economic cost has been estimated to be of the order of £3.5 billion per annum [7].

In spite of recognition at governmental level of the impact of trauma and injuries and significant advances in medical technology, evidence emerged in the 1990’s and 2000’s of a lack of improvement in the quality of trauma care [8, 9]. This was acknowledged in the pivotal NCEPOD report [10] in 2007 “Trauma: Who Cares” which led to fundamental re-shaping of trauma services in England over the last decade [11]. This has seen a regionalisation of trauma services with a hub and spoke model of trauma units feeding into a single major trauma centre in each region [12]. Evidence is emerging that this change in care has significantly improved outcomes for trauma patients in England requiring hospital treatment [13]. There has been a focus on effective, early treatment of trauma, with some benefit [14]. The method of delivery of trauma care varies between low, middle and high income countries. In many low income countries, the focus is currently on provision of effective, reliable pre-hospital care [15]. In middle and high income countries, trauma healthcare is becoming more focussed on the whole system of healthcare delivery, attempting to create a pathway for an injured patient from the scene of incident, through prehospital care and in hospital care, to rehabilitation [16, 17]. This model of major trauma centres (MTC) with a trauma network of smaller units feeding into the MTC is being more widely accepted.

Whilst the improvements in the clinical management of patient with trauma are welcome, prevention of trauma has been relatively neglected, apart from some notable exceptions such as UK legislation mandating the use of seatbelts in 1983 [18]. There is a wealth of evidence that socioeconomic deprivation is associated with increased risk of many chronic diseases, such as cardiovascular disease [19, 20]. As a result some chronic disease preventative initiatives have been targeted at deprived communities in order to reduce health inequalities [21].

Studies from other parts of the world have provided evidence of an association between low socioeconomic status and the incidence of all trauma. These studies show an association using both aggregate national or regional incidence data such as census data and individual or areal measures of social deprivation [22, 23]. There is also data showing an association between mortality and social deprivation, again using aggregate data such as census data [24, 25]. There is a relative paucity of data looking at data from individual patients in trauma registries and the association between incidence and outcome of trauma and social deprivation.

This Scotland-wide study aimed to determine whether there was any association between socioeconomic deprivation and the incidence and case fatality following trauma, in order to inform preventative interventions.

## Methods

This is a retrospective cohort study using secondary data from a national trauma registry over a 2 year period detailed in the inclusion and exclusion criteria. The data contained in the trauma registry was collected prospectively during the individual patient hospital attendance and/or admission.

### Scottish Trauma Audit Group (STAG) registry

The Scottish Trauma Audit Group (STAG) [26] is part of the Information Services Department (ISD) of NHS Scotland and has collected Scotland-wide data prospectively on all patients attending hospital for trauma since 2011. Following their presentation at hospital, patients were followed by dedicated audit staff collecting detailed information about the patients’ injury, treatment and hospital stay. The dedicated audit staff review all admissions to participating hospital Emergency Departments (ED) and Intensive Care Units (ICU) with any traumatic injury to assess if they are eligible for inclusion in the trauma registry. Patients’ injuries were objectively assessed using Abbreviated Injury Scores (AIS) 2005 descriptors [27] and Injury Severity Scores (ISS) [28] were calculated for all patients. Initial physiological observations taken on the arrival of the Scottish Ambulance Service were recorded to calculate the Revised Trauma Score (RTS) [29]. The Trauma and Injury Severity Score (TRISS) method [30] was then used to calculate an expected probability of survival for each patient using these variables. Data were then collected and entered onto a computer database centrally by STAG head office in Edinburgh. Postcodes of residence were recorded on all patients on admission. The registry is subject to monthly quality assurance exercises. Each individual patient record is validated including checks for impossible values and logic flaws between related data fields. Any electronic record failing a query is flagged and subsequently manually cross-checked against the original paper collection form. We used an extract of data from the STAG Registry to conduct a Scotland-wide retrospective cohort study.

### Inclusion and exclusion criteria

The study included adult (>16 years of age) patients with trauma attending any of the 25 major hospitals in Scotland over a 24 month period (1 January 2011 to 31st December 2012 inclusive) who either had a hospital length of stay >72 h, or died in-hospital or had initial management in a resuscitation area of the Emergency Department. Any patient arriving at a smaller hospital following trauma and requiring admission, will be transferred to one of the 25 major hospitals and included in the audit. We excluded patients presenting with isolated distal limb injury and patients over 64 years of age with isolated neck of femur fracture or pubic ramus fracture. A full list of inclusion and exclusion criteria is given in Additional file 1. We also excluded patients who were not resident in Scotland and those with missing postcode of residence for whom the Scottish Index of Multiple Deprivation (SIMD) [31] could not be calculated.

### Data definitions

The STAG registry extract provided data on age, gender, postcode of residence, mechanism of injury, Component Abbreviated Injury Scores, Combined Injury Severity Score, Predicted outcome (Ps) using TRISS method and in-hospital mortality (up to 30 days). Postcode or residence was used to determine the SIMD.

### Scottish Index of Multiple Deprivation (SIMD)

The Scottish Index of Multiple Deprivation 2012 is a measure of area level deprivation [31] and combines 38 indicators across seven domains, namely: income, employment, health, education, skills and training, housing, geographic access and crime. The level of deprivation for an individual patient is based on their domicile postcode.

The overall index is a weighted sum of the seven domain scores. The weighting for each domain is based on the relative importance of the domain in measuring multiple deprivation, the robustness of the data and the time lag between data collection and the production of the SIMD.

Prior to weighting, the domains are standardised by ranking the scores. The ranks then undergo a statistical transformation to avoid high ranks in one domain ‘cancelling out’ low ranks in another. The domain weightings used in SIMD 2012, expressed as a % of the overall weight are: current income (28 %), employment (28 %), health (14 %), education (14 %), geographic access (9 %), crime (5 %) and housing (2 %). SIMD was used to derive deciles of socioeconomic deprivation for the Scottish general population from 1 (most deprived) to 10 (least deprived). The postcode of residence of study participants was then used to allocate them to a general population decile of socioeconomic deprivation.

### Postcode sector

Postcodes are geographical areas defined by the Royal Mail in the United Kingdom for postal deliveries. In Scotland there are approximately 151,000 live postcodes. The first four alphanumeric characters of a postcode define a population on average of about 800 people. Each of these larger groups of postcodes is associated with a specific area level deprivation using the SIMD. The patient residence postcode is recorded at time of admission as part of STAG dataset.

### Injury Severity Score (ISS)

Type of Injury was classified as either blunt, such as road traffic collisions or falls from height, or penetrating, such as injuries resulting from knives or gunshot wounds. Severity of Injury was graded using the Injury Severity Score (ISS). This is a scoring system with a range of possible values from 0 to 75. It is derived from the 3 component scores of the Abbreviated Injury Score (AIS). The TRISS method [30] was then used to calculate an expected probability of survival for each patient using these variables. This was done using the 1995 probability of survival coefficients. The probability of survival was subtracted from one to give a probability of death for calculation of observed/expected death ratio.

### Population data and incidence

True population incidence of trauma requiring hospital admission was calculated using the total population of Scotland in 2011/12 as the denominator [32]. These data were obtained from National Records Scotland Office which is a government agency that provides annual updates on the total Scottish population. The numerator for incidence was the total number of episodes of trauma in the STAG registry, overall and for the various subcategories outlined in our data (age, gender, SIMD).

### Statistical analyses

Data were analysed using Stata v12 (StataCorp, College Station, TX). We used the incidence rate ratio (IRR) to provide a relative measure of the incidence rate for the various SIMD deciles, along with calculation of 95 % confidence intervals. In our study, data is reported as the most deprived decile (SIMD 1) as the control, with other deciles reported relative to this.

IRR were calculated using negative binomial regression modelling, which was tested using the Hosmer Lemeshow test. For mortality, observed/expected ratios were obtained using observed mortality in the cohort and expected deaths using Ps calculation, as outlined above. 95 % confidence intervals were calculated and statistical significance was defined as *p* < 0.05. Odds ratio for case fatality was calculated based on gender and is expressed as male equal to one with 95 % confidence intervals.

The interaction between age and SIMD Decile for case fatality was tested using a likelihood ratio. This showed a significant interaction between age and SIMD Decile with a χ^2^ = 470.21, *p* < 0.001. The interaction between gender and SIMD Decile was also tested for case fatality using a likelihood ratio. This showed a significant interaction between gender and SIMD Decile with a χ^2^ = 107.15, *p* < 0.001. The interaction between age and gender was also tested using a likliehood ratio. This did not show a significant interaction between age and gender with a χ^2^ = 2.30, *p* = 0.941.

In order to investigate the association between case-fatality and possible explanatory variables, logistic regression was undertaken. This was done using individual variables of interest first in a Univariate analysis. These were then combined in a step-wise manner to undertake a multivariate analysis. The variable of interest (outcome variable) used in each case was case fatality, with results reported as odds ratios with 95 % confidence intervals.

## Results

Over the 24 month study period, 9925 eligible patients attended Scottish hospitals for trauma. Of these, the SIMD could be calculated for 9238 (93.1 %). Of the 687 individuals with missing SIMD data, 335 (48.8 %) were non-Scottish domiciles. When comparing the demographics between those individuals in the study population with complete postcode data and those without, there was no significant difference in gender and in type and severity of trauma. However those with missing postcode were significantly younger (mean 52 versus 55 years, *p* < 0.001). Of the overall group of 9238 patients, data on type of trauma was available for all 9238 patients. Penetrating trauma accounted for 345 (3.6 %) of all trauma in our cohort.

With regards to case-fatality, outcome data was available for 100 % of 9238 eligible cases. Case fatality overall was 3.52 % (325/9238). In the blunt trauma group, the case fatality was 3.47 % (309/8893) and in the penetrating trauma group the case fatality was 4.64 % (16/345). The overall numbers of subjects in each group is shown in Table 1.

**Table 1:** Overall demographics of study dataset

		Number of subjects	Number of deaths
Total		9925	345
Age	16–24	867	21
	25–34	1069	15
	35–44	1231	18
	45–54	1650	37
	55–64	1876	43
	65–74	1346	52
	75–84	1257	87
	>85	629	72
Sex	Male	5281	203
	Female	4644	142
SIMD decile	1 (most deprived)	1536	48
	2	1153	42
	3	1002	43
	4	958	30
	5	861	35
	6	780	29
	7	779	28
	8	766	23
	9	746	32
	10 (least deprived)	657	16
	Unknown SIMD	687	19
Injury Severity Score (ISS)	Not major trauma (ISS < 16)	8435	110
	Major trauma (ISS > 15)	1487	233

Table 2 shows the incidence of trauma in the study population overall and across the subgroups of age, gender and SIMD. The incidence of all trauma increased with increasing age. The incidence of all trauma was approximately four-fold higher in the oldest age group compared with those aged 16–24 years. The same pattern was observed for blunt trauma. In contrast, there was an inverse relationship between age and the incidence of penetrating trauma, with the highest incidence in the 16–24 age group. For all types of trauma, the incidence was higher among men than women. The gender difference was most pronounced for penetrating trauma, where the incidence was ten-fold higher in men than in women.

**Table 2:** Crude annual incidence of all, blunt and penetrating trauma per 100,000 population

		All trauma % (95 % CI)	Blunt trauma % (95 % CI)	Penetrating trauma % (95 % CI)
Overall incidence		115.4 (102.5–126.3)	110.1 (98.3–121.8)	4.3 (2.4–6.3)
Age	16–24	76.4 (71.6–81.3)	67.3 (62.8–71.9)	9.1 (7.6–10.6)
	25–34	78.6 (74.1–83.1)	70.1 (65.9–74.4)	8.5 (7.2–9.7)
	35–44	87.0 (82.5–91.5)	81.1 (76.7–85.5)	5.9 (4.8–7.1)
	45–54	103.8 (99.2–108.3)	100.7 (96.2–105.2)	3.1 (2.3–3.9)
	55–64	141.7 (135.9–147.5)	140.5 (134.8–146.2)	1.2 (0.6–1.8)
	65–74	132.7 (126.1–139.3)	132.2 (125.6–138.7)	0.5 (0.1–0.9)
	75–84	203.2 (192.7–213.7)	202.4 (192.0–212.9)	0.8 (0.1–1.5)
	>85	287.9 (266.1–309.7)	287.9 (266.1–309.7)	–
Sex	Male	127.1 (124.7–129.4)	118.9 (116.6–121.2)	8.2 (7.9–8.5)
	Female	102.7 (100.6–104.9)	101.9 (99.8–104.1)	0.8 (0.5–1.0)
SIMD decile				
	1 (most deprived)	189.2 (180.6–197.8)	171.6 (163.4–180.0)	17.5 (15.2–19.7)
	2	141.1 (133.4–148.6)	133.8 (126.4–141.2)	7.2 (5.5–8.9)
	3	120.0 (113.0–127.0)	113.9 (107.0–120.7)	6.1 (4.5–7.7)
	4	110.1 (103.4–116.6)	106.7 (100.2–113.2)	3.3 (2.2–4.5)
	5	97.8 (91.5–104.0)	96.3 (90.1–102.4)	1.5 (0.7–2.3)
	6	86.4 (80.6–92.2)	84.8 (79.0–90.5)	1.7 (0.8–2.5)
	7	86.2 (80.4–92.0)	84.4 (78.6–90.1)	1.9 (1.0–2.8)
	8	84.7 (78.9–90.4)	83.1 (77.4–88.8)	1.5 (0.7–2.3)
	9	82.8 (77.1–88.5)	81.8 (76.1–87.4)	1.0 (0.4–1.6)
	10 (least deprived)	77.5 (71.8–83.2)	76.2 (70.6–81.9)	1.3 (0.5–2.1)

The incidence of all forms of trauma increased with increasing socioeconomic deprivation. For blunt and all trauma the incidence changed steadily across the socioeconomic deciles. Compared with patients in the least deprived decile of the general population, those in the most deprived decile had a 2.5 fold higher incidence of all trauma and a 2.3 fold higher incidence of blunt trauma. Compared with the most deprived decile, the least deprived had an incidence rate ratio (IRR) of 0.43 (95 % CI 0.32–0.58, *p* < 0.001) for all trauma. The association with socioeconomic deprivation was stronger among men where the least deprived decile had an IRR of 0.36 (95 % CI 0.27–0.47, *p* < 0.001) for all trauma.

The incidence of penetrating trauma also increased with increasing socioeconomic deprivation. However, the pattern differed from blunt trauma. In addition to a general trend across the least deprived 90 % of the population, there was a dramatic step-change increase in incidence in the most deprived decile. The most deprived decile of the population had an incidence of penetrating trauma which was 2.5 times higher than the next most deprived decile and 13.5 times that of the least deprived decile. Compared with the most deprived decile, the next most deprived decile had an IRR of 0.42 (95 % CI 0.16 to 1.10) and the least deprived decile had an IRR of 0.07, (95 % CI 0.01 to 0.56, *p* = 0.011).

Table 3 shows the case fatality associated with trauma overall and by subgroups of age, gender and SIMD. In general, case-fatality increased with age. There was a J-shaped curve for blunt and all trauma, whereby the case fatality was lowest in the 25–34 year age group. Compared with the age group with lowest case fatality (25–34), the oldest age group (>85) had an odds ratio of 5.21 (95 % CI 3.17 to 8.57, *p* < 0.001) for case fatality following all trauma. For all trauma, case-fatality was significantly lower in women than men (OR 0.79 (0.63 to 0.98, *p* = 0.033). There was no clear relationship between crude case-fatality and socioeconomic deprivation (Table 3).

**Table 3:** In-hospital case fatality for all trauma

		All trauma % (95 % CI)
Overall		3.52 (3.12–3.84)
Age	16–24	2.42 (1.40–2.45)
	25–34	1.40 (0.70–2.11)
	35–44	1.46 (0.79–2.13)
	45–54	2.24 (1.53–2.96)
	55–64	2.29 (1.62–2.97)
	65–74	3.86 (2.83–4.89)
	75–84	6.92 (5.52–8.33)
	>85	11.45 (8.96–13.94)
Sex	Male	3.84 (3.33–4.36)
	Female	3.06 (2.56–3.55)
SIMD	1 (most deprived)	3.12 (2.25–3.99)
	2	3.64 (2.56–4.72)
	3	4.29 (3.04–5.55)
	4	3.13 (2.03–4.24)
	5	4.07 (2.75–5.39)
	6	3.72 (2.39–5.05)
	7	3.59 (2.28–4.90)
	8	3.00 (1.79–4.21)
	9	4.29 (2.83–5.74)
	10 (least deprived)	2.44 (1.26–3.62)

*SIMD* Scottish Index of Multiple Deprivation

In the previous section a significant interaction was demonstrated between age, gender and SIMD Decile in this study population. Therefore multivariate analysis was undertaken for each of the variables of interest, stratified by each of these demographic factors. This is shown in Tables 4 and 5

**Table 4:** Multivariate analysis of variables associated with case-fatality

Variable	Univariate OR (95 % CI)	p	OR adjusted for sex only (95 % CI)	p	OR adjusted for sex & age (95 % CI)	p	OR adjusted for sex, age & SIMD decile (95 % CI)	*p*
Severity of trauma (major v non-major)	14.06 (11.13–17.78)	<0.001	15.32 (12.01–19.54)	<0.001	18.21 (14.09–23.54)	<0.001	18.17 (13.96–23.66)	<0.001
Type of injury (Penetrating Trauma)	1.41 (0.87–2.30)	0.163	1.30 (0.80–2.13)	0.292	2.48 (1.46–4.19)	0.001	2.26 (1.29–3.95)	0.004

**Table 5:** Multivariate analysis, adjusted for age, sex and SIMD decile

Variable	OR adjusted for sex, age & SIMD decile (95 % CI)	*p*
Severity of trauma (major v non-major)	18.11 (13.91–23.58)	<0.001
Type of injury (Penetrating Trauma)	2.07 (1.15–3.72)	0.016

Goodness of fit of this model was measured using Hosmer-Lemeshow

Number of groups = 8

Hosmer Lemeshow χ2 = 10.33

*P* = 0.110

In this step-wise multivariate analysis, severity of trauma (as measured by major v non major trauma) and type of injury (as measured by blunt v penetrating injury) remain as independent predictors of case fatality in this dataset.

**Fig. 1:**
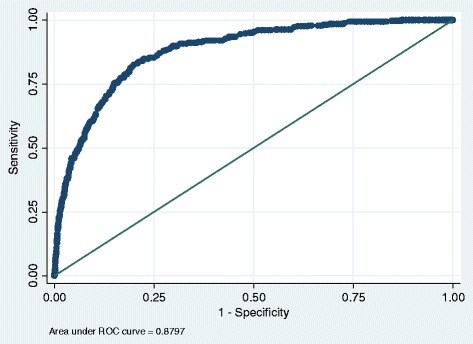
Receiver Operator Curve for final multivariate model

When the two variables of severity of trauma and type of injury are combined in a model, they both remain as independent predictors of case fatality in this group of patients. The Hosmer Lemeshow model shows a reasonable goodness of fit, but not ideal. A Receiver Operating Curve was also constructed to further examine the applicability of this model and is shown in Fig. 1. The area under the curve is 0.880 suggesting that this is a good fit for this model.

## Discussion

Our findings demonstrated an association between socioeconomic deprivation and the incidence of trauma. Patients who lived in more deprived areas were at higher risk of attending hospital for trauma.

The overall relationship with trauma masked marked differences between blunt and penetrating trauma. Whilst there was a trend across the whole socioeconomic spectrum for both, penetrating trauma showed a dramatic increase in incidence in the most deprived decile of the population who were at markedly higher risk of trauma than even the next more deprived decile. Across all types of trauma, the most deprived decile of the population were more than twice as likely to attend hospital for trauma than the least deprived decile.

Our findings are consistent with some studies from other parts of the world that have also demonstrated a higher incidence of all trauma in areas of high socioeconomic deprivation; including studies form other high income countries such as Canada [22], the United States [32–34], Norway [35], France [36], Switzerland [24], Korea [25], Sweden [37, 38], and Australia [39]. Some previous studies, conducted primarily in low income countries, have also demonstrated an association between individual-level socioeconomic deprivation status and higher incidence of trauma data [43–46] but the association with area measures of socioeconomic deprivation appears to be stronger [47, 48].

Our data did not show socioeconomic deprivation to be independently associated with case fatality following trauma, when adjusted for age and gender. Several studies [23–25, 34, 35, 37, 39, 40–42] have also shown higher population mortality from all trauma in areas of high socioeconomic deprivation. However, many of these papers were ecological studies based on aggregated national data and therefore, could not determine if higher mortality was due to higher incidence only or also to poorer survival. Our findings suggest that it is more likely that higher mortality is due to higher incidence rather than higher case-fatality.

Previous Scottish studies on socioeconomic deprivation and injury have tended to focus on specific injury types such as maxillofacial injuries [49, 50] head injuries [51] or proximal limb injuries [52, 53]. Using both the Carstairs index [54] and the SIMD [31], these studies have shown a higher incidence of these specific types of injuries in areas of high deprivation. Other studies have focused on a particular mechanism of injury. Two studies conducted in the late 1980’s showed an association between high deprivation and higher incidence of all road traffic collisions in the West of Scotland [55] and pedestrian only road traffic collisions in a single healthboard area [56]. More recently in 2013, Morrison et al published a study based on Scottish Ambulance Service data related to the postcode of the injury, rather than domicile [57]. Injury postcode was used as a proxy of domicile postcode and, thereby, to derive domicile SIMD. The investigators showed an association between high area level deprivation and higher incidence of trauma. However this study had no information on the type or severity of injury or case fatality. Furthermore, levels of employment, car ownership and disability, vary by socioeconomic status. Therefore, the extent to which injury postcode is a good proxy of domicile postcode will vary systematically by socioeconomic status, thereby introducing bias. Ours is the first Scottish study of deprivation and trauma with detailed clinical information on unselected patients across the whole of Scotland and we believe that the findings are generalisable to many other Western countries.

Our study has a number of strengths. The national trauma registry provided comprehensive, detailed information on all patients with trauma who require admission to hospital across Scotland. The data available about these patients is rich with detailed demographics, injury timeline, detailed anatomical injury information along with information about hospital admission and outcome. The data used in this study was collected prospectively specifically to look at trauma care and has a high degree of quality assurance built into the data collection system. Collection of the trauma data is undertaken by dedicated audit staff that are trained in the use of the injury severity scoring system. The data are collected across the entire country, obviating any selection bias due to geography or location of the patient. Scotland has a unique measure of social deprivation in the form of the Scottish Index of Multiple Deprivation (SIMD). This is a detailed measure of area level deprivation based on small geographic area with approximately 750 people in each unit. These geographic units are easily identifiable using domicile postcode data.

There are some limitations to the study. We were able to derive a measure of area-based socioeconomic deprivation from postcode of residence. We did not have access to individual level measures of deprivation such as income, education level and employment in different countries. However, most targeted prevention measures are likely to be targeted at deprived areas rather than deprived individuals. Our cohort comprised all patients who attended hospital following trauma but did not include patients who died prior to arrival at hospital. The proportion of trauma deaths that occur prehospital varies by country and mechanism of injury but may be up to 40 % [58, 59]. More importantly, their exclusion may introduce a systematic error into the ascertainment of severe trauma cases.

Our cohort included only those cases of trauma sufficiently severe to require hospital attendance. The majority of patients with injury or trauma do not require medical attention.

A previous British study reported that *“for every injury death there are 45 hospital episodes, 630 doctor consultations and 5000–6000 minor injuries”* [60]. Figures from the United States have shown a similar pattern with an injury death equating to 45 people hospitalised and 1300 visiting the Emergency Department [61].

The primary outcome we were able to study using the available data was in-hospital death. This is common limitation to studies on trauma. Previous studies have also tended to focus on mortality and length of stay because the data are readily accessible. Functional outcomes, such as functional capacity, are important but are more resource dependant to collect, although validated protocols exist to do this and are being more widely accepted into trauma systems [62]. Data on functional outcomes are required to evaluate the full economic impact of trauma; including lost income, benefits and social care as well as direct health care costs.

The TRISS methodology for scoring trauma related injuries and calculating survival probabilities at a population level has been in existence for some 30 years but it has attracted some criticism. One of the main criticisms levelled at the AIS 95 iteration is that it is based heavily on the original MTOS study [30] which was based on a North American population. The 1995 AIS dictionary was used in this study as the AIS 95 was the dictionary applied to the original STAG dataset at the time of data collection. This dictionary has been validated in a UK population by the Trauma Audit Research Network (TARN) group in England and Wales and, in turn, the TARN group have updated the TRISS methodology to be used with a new method of calculation and a new coefficient [63]. One of the main changes with the new coefficient is that it is based entirely on a cohort of 200,000 UK patients so the previous concerns about the North American MTOS cohort should now be negated. Since our data were based on the previous system, applying expected survival derived from a USA population to a Scottish cohort is a limitation of this study. However, for the comparisons we make between socioeconomic sub-groups in our cohort, these limitations are likely to be of less concern. Although the ISS/TRISS methodology continues to be used widely, other methods may have fewer limitations [64]. Since these data were collected in 2011/12, STAG has moved all data collection onto the 1998/2005 dictionary and also to use the TARN Ps12 coefficient.

## Conclusion

In conclusion, our study clearly demonstrates that the incidence of trauma is progressively higher with increasing levels of area level social deprivation. However there is no evidence that prognosis, as measured by in-hospital mortality, is worse among more deprived populations.

## Additional file

Additional file 1: Inclusion and exclusion criteria for Scottish Trauma Audit Group. (DOC 47 kb)
