# Supplementary material for: Association between trauma and socioeconomic deprivation: a registry-based, Scotland-wide retrospective cohort study of 9,238 patients
Source: Scand J Trauma Resusc Emerg Med. 2016 Jul 7;24:90. doi: 10.1186/s13049-016-0275-7 (PMC4937548; doi:10.1186/s13049-016-0275-7)
Supplement: Additional file 1: — Inclusion and exclusion criteria for Scottish Trauma Audit Group. (DOC 47 kb) [file 13049_2016_275_MOESM1_ESM.doc]

Additional file 1: STAG Inclusion / Exclusion Criteria

Scottish Trauma Audit Group (STAG)

**Inclusion/Exclusion criteria**

**The decision to include a patient should be based on the following points:**

1. **ALL TRAUMA PATIENTS AGED 13 AND OVER - WITH INJURIES SUSTAINED WITHIN PAST 7 DAYS**

(**DO NOT INCLUDE PATIENTS WHOSE INITIAL REASON FOR ADMISSION IS ‘SOCIAL’**)

1. **WHO FULFILL ONE OF THE FOLLOWING LENGTH OF STAY CRITERIA**

| **DIRECT TRAUMA PATIENT ADMISSIONS**  Admissions whose length of stay is at least 3 days or more – Date of attendance is counted as day ‘0’  (patients are discharged from the audit at a maximum of 30 days, or earlier if they die or when they leave an acute bed i.e. transferred to care of elderly or rehabilitation bed)  **OR**  Patients who die in hospital within 3 days of attendance    **N.B**  **Include** patients admitted to a medical ward as a surgical boarder, or under shared care of a physician and surgeon.  **Exclude** patients admitted to medical wards under the care of physician only | **TRANSFERRED TRAUMA PATIENTS (IN/OUT)**  Trauma patients transferred in/out of ED for specialist care whose combined hospital stay at both sites is 3 days or more |
| --- | --- |

1. **AND** WHOSE INJURIES DO NOT MEET THE FOLLOWING EXCLUSION CRITERIA:

| **BODY REGION**  **OR**  **SPECIFIC INJURY** | **EXCLUSIONS** |
| --- | --- |
| **HEAD** | Isolated minor head injury  (no fracture and GCS>13) |
| **FACE** | Isolated injuries to the face and #’s documented as simple or stable. |
| **THORAX** | Isolated superficial lacerations, contusions, puncture wounds/bites with no underlying injury. |
| **ABDOMEN** | Isolated superficial lacerations, contusions, puncture wounds/bites with no underlying injury. |
| **SPINE** | Pathological # |
| **PELVIS**  (incl. Acetabulum) | ≥ 65 years with pubic rami #  with or without one other isolated injury  Pathological # |
| **HIP FRACTURE** | ≥ 65 years with hip # (subcapital, intracapsular, greater trochanteric, intertrochanteric,or basal) with or without one other isolated injury.  ≥ 65 years subtrochenteric # or proximal femoral #, if treated as a hip #  Pathological #  Isolated dislocations/ prosthetic dislocations |
| **BODY REGION**  **OR**  **SPECIFIC INJURY** | **EXCLUSIONS** |
| **FEMUR** | Proximal femoral shaft #’s treated as hip #’s  Pathological # Periprosthetic # |
| **LIMB - LOWER**  **(KNEE and BELOW)**  **(EXCEPT Feet/Toes)** | Any closed unilateral injury (in isolation) |
| **LIMB – UPPER**  **(EXCEPT Hands/Fingers)**  **N.B Scapula and Clavicle are also defined as upper limb injuries** | Any closed unilateral injury (in isolation) |
| **FOOT OR HAND:**  **JOINT OR BONE** | Any # &/or dislocations, even if open &/or multiple (in isolation)  **N.B Only include - Crush or amputation** |
| **FINGERS OR TOES** | All |
| **NERVE** | All nerve injuries, single or multiple (except injury to sciatic, facial, femoral or cranial nerve) |
| **MUSCLE** | All |
| **VESSEL** | Intimal tear or superficial laceration or perforation to any limb vessel (however, include all injuries to femoral, neck, facial, cranial, thoracic or abdominal vessels. Transection or major disruption of any other vessel) |
| **SKIN** | Simple skin lacerations, contusions, puncture wounds and bites with no underlying injury.  Minor de-gloving injury (if not in AIS dictionary)  Re: Major de-gloving injury (see AIS dictionary for guidance i.e. If not in dictionary then not scoreable) |
| **BURN** | All |
| **INHALATION** | All |
| **AMPUTATION/CRUSH** | Fingers/toes |
| **ASPHYXIA**  **(e.g. attempted hanging)** | None |
| **DROWNING** | None |
| **ELECTRICAL** | None |
| **EXPLOSION** | None |
| **FROSTBITE** | Superficial frostbite (except deep; full thickness; multi body sites e.g. fingers, toes, ears) |
| **HYPOTHERMIA** | >31°  Or Hypothermia in isolation |

**If you are uncertain whether a patient should be included in the audit, please contact the central office team for further guidance**
